# Mass spectrometry-based intraoperative tumor diagnostics: a letter in reply

**DOI:** 10.2144/fsoa-2019-0037

**Published:** 2019-08-01

**Authors:** Isabelle Fournier, Michel Salzet

**Affiliations:** 1 Réponse Inflammatoire et Spectrométrie de Masse (PRISM), Université de Lille, Inserm U1192 - Laboratoire Protéomique, F-59655 Villeneuve d’Ascq Cedex, France

**Keywords:** cancer, guided surgery, intraoperative tumor diagnostics, real-time diagnosis, SpiderMass, surgery, tumor margin

We wish to offer our perspective and expertise concerning the review article entitled ‘mass spectrometry-based intra-operative tumor diagnosis’ written by Lorena Hänel *et al.* in *Future Science OA*. We were struck reading this article by the fact that the authors did not properly address the state-of-the-art of the field in their review and were not addressing all the technologies developed over the past years to address intraoperative tumor diagnostic by MS. As a matter of fact, the authors fail to mention the SpiderMass technology. Therefore, they provide inadequate and not up-to-date citation on this discussed topic. In 2014, PRISM lab (Lille, France) introduced the water-assisted laser desorption/ionization MS (SpiderMass) and filed the patent (PCT/IB2015/057301) [1]. Similar to iKnife [2] and MassSpec Pen [3], the SpiderMass constitutes a micro-sampling probe, transfer line and MS analyzer. The system is based on laser ablation in the infrared (IR) wavelength tuned to excite the most intense vibrational band (O–H) of water molecules, called water-assisted laser ionisation/desorption process. The water constitutes an average 70–80% of the tissues in the human body, and can act as an endogenous matrix and provide analyte ionization as in conventional MALDI [4]. After the laser ablation, ions are collected using the MS vacuum via polymeric tubing positioned just above the analyzed tissue and connected directly to the inlet of the MS source. In this way, the aerosol is delivered to the analyzer, without the need of post-ionization device. As a result, the molecular profiles of ablated tissue are acquired, and can be further compared with a databank containing recorded *ex vivo* molecular profiles of a cohort of biopsies for example. One of the major advantages of the SpiderMass system is its low invasiveness and painless sampling, tissues being reversibly dehydrated. Another advantage and specific characteristic of SpiderMass is the very low fragmentation observed in the spectra and the possible operation in both positive and negative ion modes with good sensitivity. The system was successfully applied for cancer biopsies analysis *ex vivo* and then was shown for allowing real-time analysis of human skin *in vivo* as presented for the first time in 2015 at the ASMS conference and then published in 2016 [5]. The publication evidently shows the first use of a laser-based system coupled to a mass spectrometer for *in vivo* analysis and its potential to be used in intraoperative diagnostics. Over the past few years, the system was used to analyze genetically modified macrophage cell lines [6] as well as intact proteins. The latest publication in 2018 showed the analysis of dog sarcoma samples *ex vivo*, allowing for correct classification (97% of specificity) of tumor type and grading [7]. Furthermore, the system demonstrated its applicability for *in vivo* intraoperative analysis at the surgery room in a veterinary clinic on dog patients [7]. A recent publication in Cancer Cell by Dr Emily Z Keung and Christina L Roland, from the Department of Surgery Oncology of The University of Texas MD Anderson Cancer Center, acknowledged this work and mentioned the SpiderMass system as a step forward in the right direction for molecular cancer diagnostics [8]. The aforementioned examples clearly show that the PRISM Laboratory with its SpiderMass technology is one of the important research groups developing MS-based intraoperative diagnostics [9].

## References

[1] Salzet M, Fournier I, Focsa C, Ziskind M, Fatou B, Wisztorski M Device for real-time *in vivo* molecular analysis. Google Patents (2017). https://patents.google.com/patent/CA2961491A1/en?q=salzet&inventor=fournier&oq=fournier+and+salzet

[2] Schafer KC, Denes J, Albrecht K *In vivo*, *in situ* tissue analysis using rapid evaporative ionization mass spectrometry. Angew. Chem. Int. Ed. Engl. 48(44), 8240–8242 (2009). 19746375 10.1002/anie.200902546

[3] Zhang J, Rector J, Lin JQ Nondestructive tissue analysis for *ex vivo* and *in vivo* cancer diagnosis using a handheld mass spectrometry system. Sci. Transl. Med. 9(406), eaan3968 (2017). 28878011 10.1126/scitranslmed.aan3968 PMC5830136

[4] Fournier I, Fatou B, Wisztorski M, Focsa C, Ziskind M, Salzet M Development of a novel instrument for *ex-vivo* and *in-vivo* real-time analysis. J. Biotechnol. (208), S10 (2015).

[5] Fatou B, Saudemont P, Duhamel M *et al* Real time and *in vivo* pharmaceutical andenvironmental studies with 944 SpiderMass instrument. J. Biotechnol. 281, 61–66 (2015). 10.1016/j.jbiotec.2018.06.339 29908205

[6] Fatou B, Saudemont P, Leblanc E *Invivo* real-time mass spectrometry for guided surgery application. Sci. Rep. 6, 25919 (2016). 27189490 10.1038/srep25919 PMC4870577

[7] Fatou B, Ziskind M, Saudemont P Remote atmospheric pressure infrared matrix-assisted laser desorption-ionization mass spectrometry (remote IR-MALDI MS) of proteins. Mol. Cell. Proteomics 17(8), 1637–1649 (2018). 29653959 10.1074/mcp.TIR117.000582 PMC6072536

[8] Saudemont P, Quanico J, Robin Y-M Real-time molecular diagnosis of tumors using water-assisted laser 942 desorption/ionization mass spectrometry technology. Cancer Cell 34, 840.e4–851.e4 (2018). 30344004 10.1016/j.ccell.2018.09.009

[9] Keung EZ, Roland CL Accurate and reproducible diagnosis of canine soft tissue sarcoma using mass spectrometry: a step in the right direction. Cancer Cell 34(5), 697–699 (2018). 30423292 10.1016/j.ccell.2018.10.013 PMC7643712

[10] Hanel L, Kwiatkowski M, Heikaus L, Schluter H Mass spectrometry-based intraoperative tumor diagnostics. Future Sci. OA 5(3), FSO373 (2019). 30906569 10.4155/fsoa-2018-0087 PMC6426168

